# Diagnostic utility of quantitative interferon-gamma release assays in elderly patients with tuberculosis

**DOI:** 10.1128/spectrum.02763-25

**Published:** 2026-02-10

**Authors:** Yoshikazu Mutoh, Yusuke Minato, Yuya Kawamoto, Shogo Hanai, Takumi Umemura, Hiroko Suzuki, Yuta Nishina, Kaho Hiramitsu, Seiya Ichihara, Satoshi Hagimoto, Jun Fukihara, Hajime Sasano, Kensuke Kataoka, Tomoki Kimura, Yohei Doi

**Affiliations:** 1 Department of Infectious Diseases, Tosei General Hospital 37091, Seto, Aichi, Japan; 2 Department of Microbiology, Fujita Health University School of Medicine 89305, Toyoake, Aichi, Japan; 3 Division of Anti-Infective Drug Discovery, Center for Infectious Disease Research, Fujita Health University 12695, Toyoake, Aichi, Japan; 4 Department of Infectious Diseases, Fujita Health University School of Medicine 89305, Toyoake, Aichi, Japan; 5 Department of Infection Control team, Tosei General Hospital 37091, Seto, Aichi, Japan; 6 Department of Respiratory Medicine and Allergy, Tosei General Hospital 37091, Seto, Aichi, Japan; University of Kentucky, Lexington, Kentucky, USA

**Keywords:** tuberculosis, elderly, interferon-gamma release assay, QuantiFERON-TB Gold Plus, T-SPOT.TB, diagnosis

## Abstract

Evidence regarding the diagnostic value of quantitative interferon-gamma release assay (IGRA) results in elderly populations is limited, and large-scale data for QuantiFERON-TB Gold Plus (QFT-Plus) are scarce. We evaluated QFT-Plus and T-SPOT.TB (T-SPOT) for distinguishing active tuberculosis (ATB) from latent infection (LTBI) in elderly individuals in Japan, a super-aged country. We conducted a retrospective, cross-sectional diagnostic accuracy study of patients ≥65 years who underwent IGRA testing between 2015 and 2024 at two hospitals: a tuberculosis referral center (QFT-Plus and T-SPOT) and a tertiary hospital (T-SPOT only). ATB was defined as microbiologically confirmed TB. Quantitative IGRA values were compared between ATB and LTBI in all patients and in IGRA-positive subsets. Receiver operating characteristic (ROC) curves assessed discriminatory performance. Among 10,745 elderly patients (ATB: *n* = 310; LTBI: *n* = 1,158), values showed substantial overlap. For T-SPOT, the area under the curves (AUCs) improved at Tosei General Hospital (TGH) (ESAT-6: 0.679, CFP-10: 0.670) in IGRA-positive cases. In contrast, all-patient AUCs at Fujita Health University Hospital (FHUH) were low (ESAT-6: 0.367, CFP-10: 0.362), demonstrating an inverse association, though they improved (ESAT-6: 0.607 and CFP-10: 0.554) in IGRA-positive cases. For QFT-Plus, all-patient AUCs were low (TB1 antigen: 0.462, TB2 antigen: 0.470), but improved in the IGRA-positive cohort (TB1 antigen: 0.630, TB2 antigen: 0.645). The optimal quantitative cutoffs in IGRA-positive cases provided modest diagnostic accuracy. In elderly individuals, quantitative IGRA values alone have limited ability to distinguish ATB from LTBI, but QFT-Plus and T-SPOT show modest improvement in IGRA-positive cases. Although not suitable as a stand-alone diagnostic, quantitative IGRA may assist risk stratification and decision-making in selected scenarios.

**IMPORTANCE:**

Tuberculosis remains a major health concern in aging societies, such as Japan, where most patients are elderly adults with impaired immune function. Interferon-gamma release assays (IGRA) are widely used for detecting infection, but the role of their quantitative values in differentiating active tuberculosis from latent tuberculosis infection has been uncertain. Our study is the first to evaluate the quantitative performance of the latest QuantiFERON-TB Gold Plus and T-SPOT.TB specifically in elderly patients, across both a tuberculosis referral hospital and a university hospital. Although absolute separation between active and latent disease was not achieved, we found that, in test-positive individuals, active cases tended to yield higher values, particularly with T-SPOT.TB. This indicates that quantitative information, when interpreted within the clinical context, can assist physicians in assessing risk and guiding further diagnostic steps, offering practical value for improving decision-making in the care of vulnerable elderly patients.

## INTRODUCTION

Tuberculosis (TB), the infectious disease caused by *Mycobacterium tuberculosis* (Mtb), affects approximately 10.8 million individuals and claims 1.25 million lives annually worldwide, making it one of the most prevalent infectious diseases today (1). Latent tuberculosis infection (LTBI) is estimated to affect nearly one-quarter of the global population (2). Since the COVID-19 pandemic, the incidence of both active TB (ATB) and LTBI has shown a concerning increase. In Japan, approximately 10,000 TB cases are reported each year, with more than 70% occurring in elderly individuals. The mortality rate among this population has exceeded 20% in recent years, underscoring TB as a persistent public health menace in the super-aged society (3).

Interferon-gamma release assay (IGRA) was first introduced to the U.S. Food and Drug Administration in 2001 as QuantiFERON-TB, which measures interferon-gamma release in response to purified protein derivative (4). Compared with the tuberculin skin test, IGRA requires only a single visit, is performed on blood samples, and is not affected by prior BCG vaccination or infection by most non-tuberculous mycobacteria.

Over the past two decades, IGRAs have been widely adopted globally as an immunodiagnostic tool to detect Mtb infection, including LTBI, particularly in individuals suspected of TB, those in close contact with ATB patients, or those undergoing immunosuppressive therapy. Currently available IGRAs include the QuantiFERON test (QFT), which measures IFN-γ levels in antigen-stimulated cell supernatants via enzyme-linked immunoassay, and T-SPOT.TB (T-SPOT), which enumerates IFN-γ-producing memory T cells using the enzyme-linked immunospot (ELISpot) method.

Both assays demonstrate robust diagnostic performance in general populations. A prior meta-analysis reported the sensitivity and specificity of QFT and T-SPOT as 90.2% and 91%, and 98% and 98%, respectively, for detecting LTBI (5). Among people living with HIV, sensitivity declines to 66.3% for QFT and 60.4% for T-SPOT.TB (6). In elderly individuals, however, evidence regarding IGRAs’ diagnostic value remains equivocal. Age-associated immune senescence, including reduced T-cell responsiveness and impaired interferon-gamma secretion, has been implicated in higher rates of indeterminate and false-negative results (7). In patients with active tuberculosis, Yamasue et al. reported a twofold increase in the likelihood of false-negative IGRA outcomes with advanced age (8), and Kobashi et al. observed significantly more indeterminate QFT results in older adults (9). Conversely, other studies found no significant association between age and IGRA positivity, leaving the clinical utility of IGRA in elderly populations unresolved (10). Despite the widespread availability of IGRAs, few studies have rigorously assessed their utility in diagnosing active TB specifically in older adults.

Moreover, while IGRAs offer quantitative measures of immune response, their numerical values have traditionally been dismissed as diagnostically uninformative—particularly in distinguishing ATB from LTBI—due to substantial overlap between patient groups. However, this prevailing assumption may reflect a lack of rigorous investigation rather than definitive evidence of irrelevance, especially in aging populations where immune responsiveness is often attenuated. To date, few studies have systematically examined whether quantitative IGRA values differ meaningfully between microbiologically confirmed ATB and smear- or culture-negative cases. A more granular analysis of IGRA distributions, particularly at higher values and in relation to clinical features, may reveal diagnostic signals currently overlooked in routine interpretation. In a country like Japan, where TB disproportionately affects older adults, re-examining both the qualitative and quantitative dimensions of IGRA interpretation is essential for refining diagnostic precision and enhancing risk stratification.

Against this backdrop, we conducted a two-center, real-world analysis to clarify both the qualitative and quantitative diagnostic utilities of IGRAs in elderly individuals, in order to refine risk stratification and clinical decision-making in Japan’s super-aged society.

## MATERIALS AND METHODS

### Subjects and study design

We conducted a retrospective, cross-sectional diagnostic accuracy study among IGRA-examined elderly patients (>65 years old) between January 2015 and December 2024 at Tosei General Hospital (TGH) and Fujita Health University Hospital (FHUH).

TGH is a municipal referral hospital with around 630 inpatient beds authorized for TB hospitalization. FHUH is an academic tertiary referral hospital with 1,376 inpatient beds, but it is not a designated admitting facility for TB patients.

Clinical data, including demographic, microbiological, and laboratory variables, were retrospectively extracted from electronic medical records of elderly patients who underwent IGRA testing, regardless of clinical testing indication, and had respiratory specimens—including gastric aspirates and bronchoscopic specimens—submitted for mycobacterial evaluation (Ziehl-Neelsen staining, nucleic acid amplification testing, or culture) within 90 days of IGRA testing.

In cases where multiple IGRA tests were performed for the same patient, the test closest to the date of respiratory specimen collection for TB testing was used for primary analysis, while the highest value within the same episode was retained for quantitative evaluation; episodes separated by more than 90 days were considered distinct and analyzed independently. ATB was defined as microbiologically confirmed TB or positive nucleic acid amplification tests from respiratory specimens. LTBI was defined as cases with positive IGRA results without microbiological confirmation of TB, regardless of clinical or radiological suspicion or anti-TB treatment initiation.

### Interferon-gamma release assays

Two IGRAs were evaluated in this study: the QuantiFERON-TB Gold Plus (QFT-Plus; Qiagen, Venlo, the Netherlands) and the T-SPOT (Oxford Immunotec, Abingdon, UK). Both assays were performed at TGH, whereas only the T-SPOT was employed at FHUH.

#### QFT-Plus

For the QFT-Plus assay, 1 mL of peripheral blood was collected into each of four tubes containing TB1 antigen (ESAT-6 and CFP-10), TB2 antigen (ESAT-6, CFP-10, and TB7.7), a negative control (Nil), and a positive control (Mitogen). Following incubation at 37°C for 16–24 hours, plasma IFN-γ concentrations were measured by enzyme-linked immunosorbent assay (ELISA). A cutoff of ≥0.35 IU/mL was considered positive, in line with the manufacturer’s instructions. Values below the lower detection limit (0.5 IU/mL) were assigned as 0.0 IU/mL, and values exceeding the upper detection limit (10.0 IU/mL) were recorded as 10.0 IU/mL.

#### T-SPOT

For the T-SPOT assay, peripheral blood mononuclear cells (PBMCs) were isolated from whole blood, washed, and plated. Cells were stimulated with ESAT-6 (panel A) and CFP-10 (panel B), and the number of IFN-γ-producing T cells was enumerated by ELISpot assay. Results were interpreted as positive if either panel yielded ≥8 spot-forming cells, negative if both panels had ≤4 spots, and indeterminate if both panels yielded 5–7 spots. In accordance with the institutional protocol at TGH, results exceeding the upper limit of 50 spots were recorded as 50.

Both assays were performed strictly according to the manufacturer’s instructions.

### Outcomes

The primary objective of this study was to evaluate the diagnostic performance of quantitative IGRA in elderly patients with microbiologically confirmed ATB, and to assess the discriminatory power of quantitative IGRA values in differentiating ATB from LTBI. The secondary objectives were to compare positivity rates and quantitative result distributions between QFT-Plus and T-SPOT based on institutional testing protocols; to analyze IGRA response patterns in hospitalized ATB patients; and to assess the qualitative diagnostic utility of IGRA using standard manufacturer-defined positivity cutoffs in this population.

### Statistical analysis

Categorical variables were expressed as counts and percentages, and continuous variables were presented as mean ± standard deviation (SD) or median with interquartile range (IQR), as appropriate. Normality was assessed using the Shapiro–Wilk test. For group comparisons, categorical variables were analyzed using the χ² test or Fisher’s exact test. Continuous variables were compared using the Mann–Whitney U test for non-normally distributed data, and the Student’s *t*-test was additionally applied when mean differences were evaluated. For both IGRA methods, sensitivity, specificity, positive predictive value, and negative predictive value were calculated. Positive and negative likelihood ratios, as well as odds ratios, were also computed. Receiver operating characteristic (ROC) curves and violin plots were generated to visualize IGRA distributions. All statistical tests were two-tailed, and a *P* value of <0.05 was considered statistically significant. Statistical analysis was performed using R statistical software (version 4.5.0; R Core Team, 2025).

Study information was disclosed in a continuously updated repository on the institutional website, and patients could request exclusion at any time. Any opt-out requests were formally documented and handled by the study team.

## RESULTS

**Fig 1:**
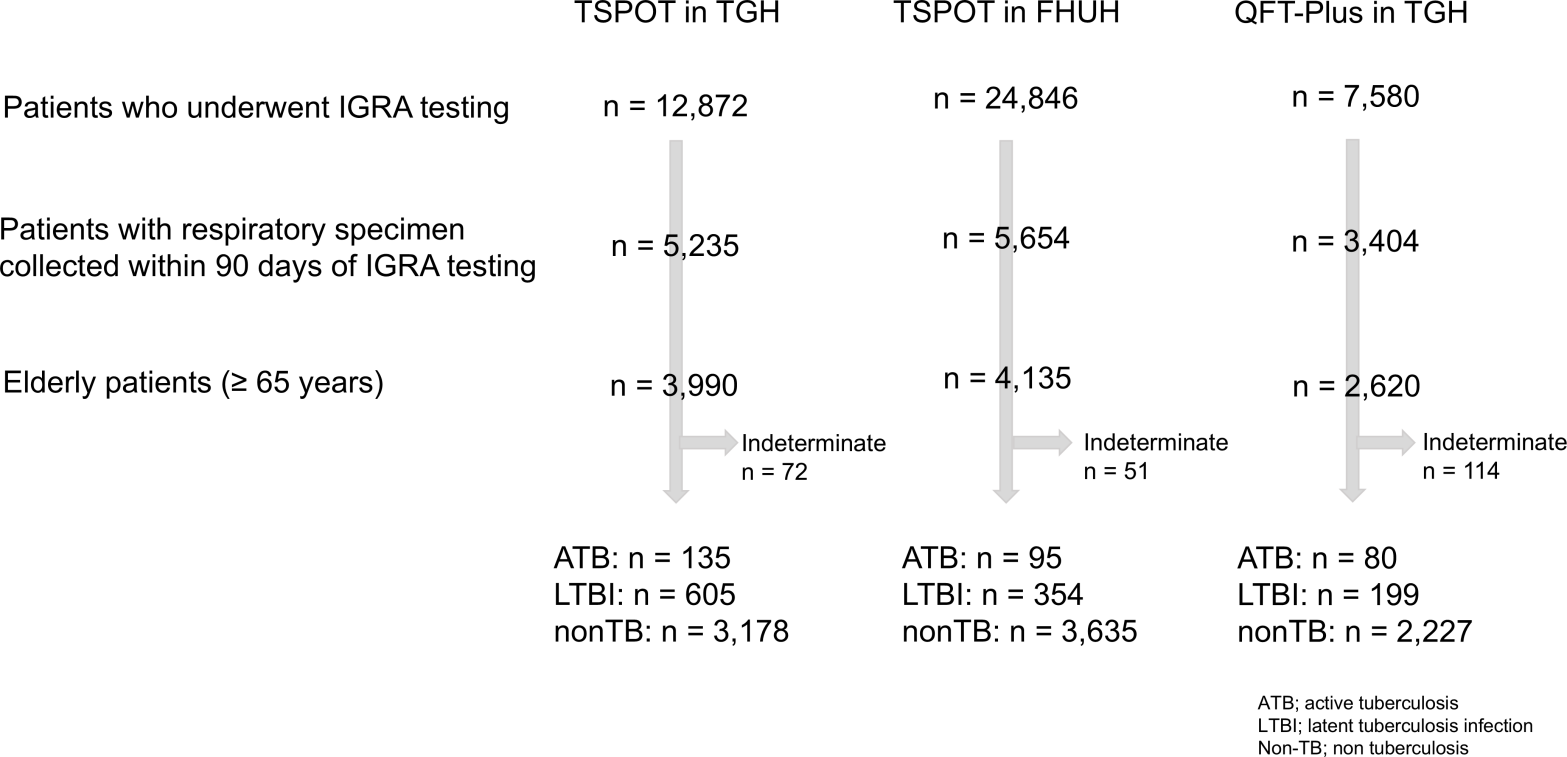
Patient flow and categorization across IGRA testing facilities. Patients who underwent IGRA testing at TGH and FHUH were screened. Subsequent stratification was performed based on respiratory specimen collection and age (≥65 years). The final cohort was classified into ATB, LTBI, and non-TB groups for each IGRA method. Indeterminate results are shown separately.

Throughout the study period, a total of 45,298 patients underwent IGRA testing. Of these, 12,872 patients received T-SPOT testing at TGH, 24,846 at FHUH, and 7,580 underwent QFT-Plus testing at TGH. The numbers of patients diagnosed with ATB and LTBI in each group were as follows—TGH T-SPOT: 135 (3.4%) and 605 (15.2%); FHUH T-SPOT: 95 (2.3%) and 354 (8.6%); TGH QFT-Plus: 80 (3.1%) and 199 (7.6%), respectively (Fig. 1).

In the T-SPOT cohort, the median age was 76.0 years [IQR: 71–82] at TGH and 75.0 years [IQR: 71–80] at FHUH, with females comprising 38.0% and 35.5%, respectively. In the QFT cohort at TGH, the median age was 77.0 [IQR: 73–83] years, and females accounted for 40.1%. Among patients with ATB, the proportion of patients testing IGRA-positive differed across cohorts: 84.4% in TGH-T-SPOT, 53.7% in FHUH-T-SPOT, and 72.5% in TGH-QFT (Table 1).

**TABLE 1:** Baseline characteristics of elderly patients undergoing IGRA testing at two institutions^*a*^

	T-SPOT		QFT-Plus
	TGH *n* = 3,918	FHUH *n* = 4,084	TGH *n* = 2,506
Median age, y [IQR]	76.0 [71–82]	75.0 [71–80]	77.0 [73–83]
Female, *n* (%)	1,488 (38.0)	1,448 (35.5)	1,005 (40.1)
ATB patients, *n* (%)	135 (3.4)	95 (2.3)	80 (3.2)
Positive, *n* (%)	114 (84.4)	51 (53.7)	58 (72.5)
Negative, *n* (%)	13 (9.6)	38 (40.0)	22 (27.5)
Borderline, *n* (%)	8 (5.9)	6 (6.3)	0 (0.0)

^*a*^ Data are shown separately for T-SPOT performed at TGH and FHUH, and for QFT-Plus performed at TGH. Values are presented as numbers (%) unless otherwise indicated. Age is shown as median [IQR]. ATB, active tuberculosis; LTBI, latent tuberculosis infection; non-TB, no evidence of tuberculosis.

**Fig 2:**
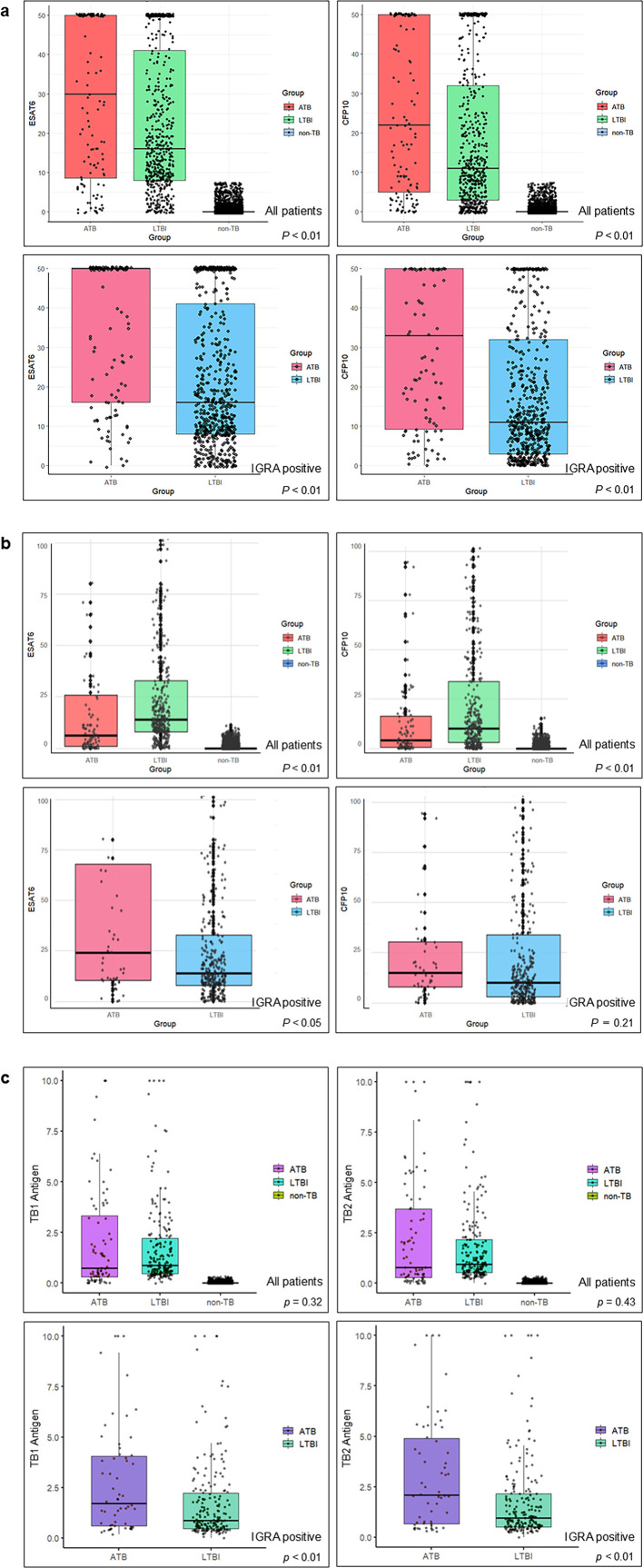
(**a**) Distribution of T-SPOT.TB quantitative results (ESAT-6 and CFP-10) in ATB, LTBI, and non-TB groups at TGH. Box plots show results for ATB, LTBI, and non-TB groups at TGH. Comparisons are displayed for all patients and for IGRA-positive patients only. *P* values indicate comparison between ATB and LTBI groups. (**b**) Distribution of T-SPOT.TB quantitative results (ESAT-6 and CFP-10) in ATB, LTBI, and non-TB groups at FHUH. Box plots show results for ATB, LTBI, and non-TB groups at FHUH. Comparisons are displayed for all patients and for IGRA-positive patients only. *P* values indicate comparison between ATB and LTBI groups. (**c**) Distribution of QFT-Plus quantitative results (TB1 and TB2 antigen values) in ATB, LTBI, and non-TB groups at TGH. Box plots show results for ATB, LTBI, and non-TB groups. Analyses are displayed for all patients and for IGRA-positive patients only. *P* values indicate comparison between ATB and LTBI groups.

Table 2 presents the mean and median quantitative IGRA results stratified by ATB, LTBI, and non-TB groups, and Fig. 2 illustrates the box plots for each assay. At TGH, both T-SPOT (ESAT-6 and CFP-10) measures in the ATB group showed significantly higher median values compared with the LTBI group (*P* < 0.01 for both). Among T-SPOT-positive patients only, the ATB group showed significantly higher median values for both antigens (*P* < 0.01 for each). At FHUH, the LTBI group had significantly higher median values for both T-SPOT antigens (*P* < 0.01 for both). However, among T-SPOT-positive patients only, the median value of CFP10 did not show a significant difference (*P* = 0.21). In QFT testing, neither TB1 nor TB2 antigen values showed significant differences compared with the LTBI group (*P* = 0.32 for TB1 and *P* = 0.43 for TB2). Among the QFT-positive patients only, the ATB group showed significantly higher median values for both antigens (*P* < 0.01 for each) (Tables 2 and 3; Fig. 2a through c).

**TABLE 2:** Quantitative T-SPOT.TB results (ESAT-6 and CFP-10) in elderly patients with ATB, LTBI, and non-TB groups^*a*^

	Group	ATB median [IQR]	LTBI median [IQR]	non-TB median [IQR]	*P* value (ATB vs LTBI)
TGH					
All	ESAT6	30 [8.5–50]	16 [8–41]	0 [0–0]	*P* < 0.01
	CFP10	22 [5–50]	11 [3–32]	0 [0–0]	*P* < 0.01
IGRA-positive	ESAT6	50 [16–50]	16 [8–41]		*P* < 0.01
	CFP10	33 [9.25–50]	11 [3–32]		*P* < 0.01
FHUH					
All	ESAT6	6 [1.0–26.0]	14 [8.0–32.8]	0 [0–0]	*P* < 0.01
	CFP10	4 [0.5–16.5]	10 [3.0–34.0]	0 [0–0]	*P* < 0.01
IGRA-positive	ESAT6	24 [10.5–68]	14 [8–32.8]		*P* < 0.0*5*
	CFP10	15 [8–30.5]	10 [3.0–34.0]		*P* = 0.21

^*a*^ Data are presented as median [IQR], stratified by institution (TGH and FHUH). Analyses are shown for all patients and for IGRA-positive patients only. ATB, active tuberculosis; LTBI, latent tuberculosis infection; non-TB, no evidence of tuberculosis.

**TABLE 3:** Quantitative QFT-Plus results (TB1 and TB2 antigen values) in elderly patients with ATB, LTBI, and non-TB groups at TGH^*a*^

	Group	ATB median [IQR]	LTBI median [IQR]	non-TB median [IQR]	*P* value (ATB vs LTBI)
All	TB1 antigen	0.73 [0.31–3.33]	0.86 [0.47–2.22]	0 [0–0]	*P* = 0.32
	TB2 antigen	0.78 [0.27–3.70]	0.95 [0.52–2.16]	0 [0–0]	*P* = 0.43
IGRA-positive	TB1 antigen	1.72 [0.61–4.07]	0.86 [0.47–2.22]		*P* < 0.01
	TB2 antigen	2.1 [0.67–4.90]	0.95 [0.52–2.16]		*P* < 0.01

^*a*^ Data are presented as median [IQR], stratified by institution (TGH). Analyses are shown for all patients and for IGRA-positive patients only. ATB, active tuberculosis; LTBI, latent tuberculosis infection; non-TB, no evidence of tuberculosis.

**Fig 3:**
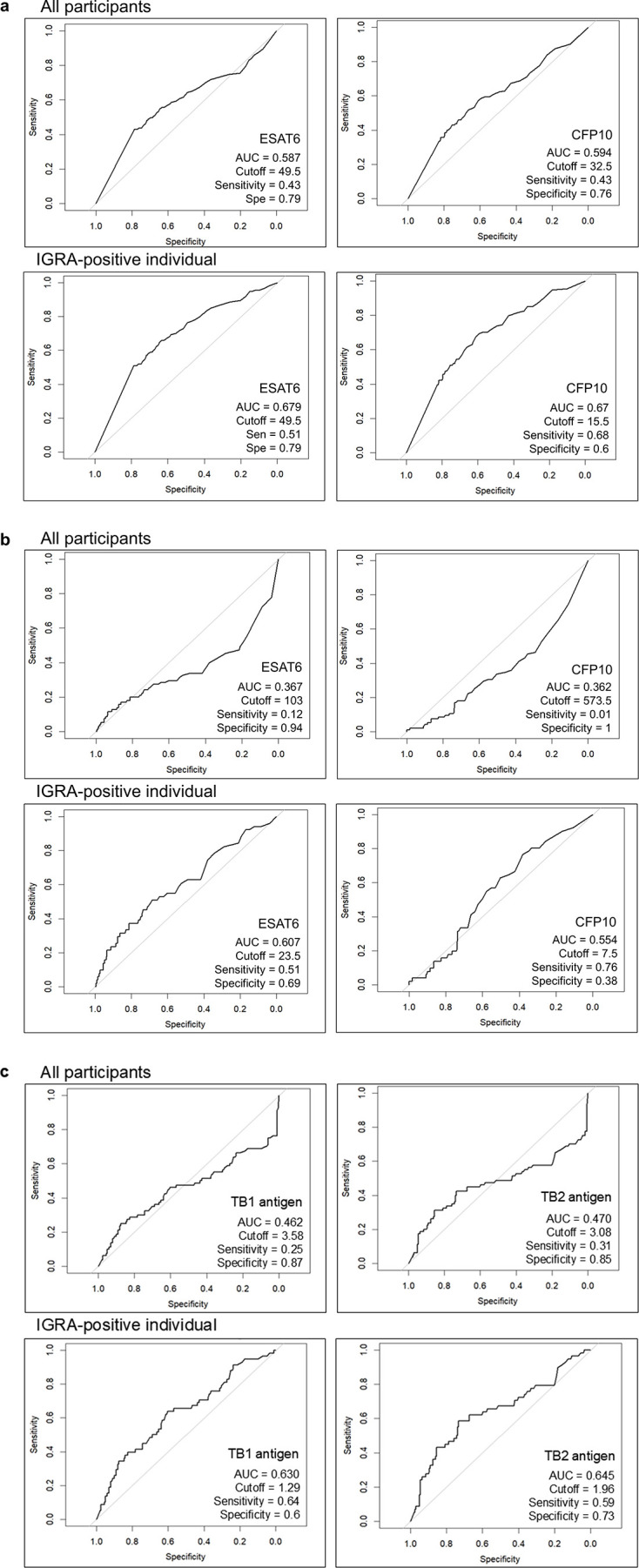
ROC curves of T-SPOT.TB and QFT-Plus results in elderly patients at TGH and FHUH. Curves are shown for ESAT6 and CFP10 spot counts (T-SPOT) and TB1 and TB2 antigen values (QFT-Plus) in all participants and in IGRA-positive individuals. For each curve, AUC, optimal cutoff, sensitivity, and specificity are indicated. The top row represents all participants, and the bottom row represents IGRA-positive individuals. (**a**) ROC curves of T-SPOT.TB results in elderly patients at TGH. (**b**) ROC curves of T-SPOT.TB results in elderly patients at FHUH. (**c**) ROC curves of QFT-Plus results in elderly patients at TGH.

For T-SPOT at TGH, ROC analysis yielded area under the curve (AUC) values of 0.587 for ESAT-6 and 0.594 for CFP-10, indicating low discriminatory performance. When the analysis was restricted to T-SPOT-positive patients at TGH, the AUC values improved slightly to 0.679 for ESAT6 and 0.670 for CFP10, suggesting modest discriminatory ability. The cut-off value was 49.5 for ESAT6 (sensitivity, 0.51; specificity, 0.79) and 15.5 for CFP10 (sensitivity, 0.68; specificity, 0.6). (Fig. 3a).

For T-SPOT at FHUH, the AUC values in the overall cohort were lower (0.367 for ESAT-6 and 0.362 for CFP-10), showing an inverse trend. When the analysis was restricted to T-SPOT-positive patients at FHUH, the corresponding AUC values were 0.607 for ESAT6 and 0.554 for CFP10, indicating low discriminatory performance (Fig. 3b).

For QFT-Plus at TGH, the overall AUC values were 0.462 for TB1 and 0.470 for TB2, indicating poor discriminatory performance. When the analysis was limited to QFT-positive patients, the AUC values improved to 0.630 for TB1 and 0.645 for TB2, suggesting modest discriminatory ability. The cut-off value was 1.29 for TB1 (sensitivity, 0.64; specificity, 0.60) and 1.96 for TB2 (sensitivity, 0.59; specificity, 0.73) (Fig. 3c).

## DISCUSSION

We evaluated the quantitative performance of IGRA testing among elderly individuals from two hospitals with and without a microbiological diagnosis of TB focus in Japan. To our knowledge, this is the first and largest real-world study to assess IGRA in this population, and the first to examine quantitative differences between ATB and LTBI using QFT-Plus, the fourth-generation QFT assay. In the overall cohort, neither T-SPOT nor QFT demonstrated strong discriminatory performance for differentiating ATB from LTBI. In addition to quantitative values, we also evaluated the qualitative diagnostic performance of IGRA using standard manufacturer-defined cutoffs. Consistent with previous studies, qualitative positivity alone showed limited ability to distinguish ATB from LTBI in elderly individuals. However, when the analysis was restricted to IGRA-positive patients, both assays showed modest discriminatory ability. These findings highlight the importance for clinicians to recognize the limitations of IGRA in distinguishing ATB from LTBI and to interpret results in conjunction with other clinical information, such as sputum smear microscopy, chest radiography, and patient symptoms.

In Japan, QuantiFERON TB-Gold was approved as the first IGRA in 2005, followed by approval of QuantiFERON TB-Gold In-Tube and QFT-Plus in 2009 and 2018, respectively. On the other hand, T-SPOT was approved in 2012. Currently, both QFT-Plus and T-SPOT are used widely. The principles of these IGRAs differ. For QFT-Plus, peripheral venous blood is collected and incubated *in vitro* with three Mtb-specific antigens: ESAT-6, CFP-10, and TB7.7. Following antigenic stimulation, IFN-γ released by lymphocytes is quantified in the plasma using ELISA (11). In contrast, for T-SPOT, PBMCs are isolated from peripheral blood and added to a 96-well plate pre-coated with anti-human IFN-γ antibodies. After the addition of specific TB antigens (ESAT-6 and CFP-10), the cells are incubated for approximately 20 hours. The number of IFN-γ-secreting cells is then counted (12). T-SPOT standardizes the number of PBMCs in each assay, allowing for a more direct comparison of antigen-specific immune responses between individuals. In contrast, QFT-Plus measures the overall amount of IFN-γ released in whole blood, which, in principle, depends on the number of immune cells present in the sample. Therefore, the absolute IFN-γ value in QFT-Plus is generally considered unreliable for distinguishing between LTBI and ATB. Nevertheless, some studies have reported an apparent association between the activity of tuberculosis infection and the magnitude of IFN-γ secretion by lymphocytes, although substantial overlap in quantitative T-SPOT results between ATB and LTBI has been consistently observed (13). For example, Ma et al. demonstrated that T-SPOT scores in ATB and old TB were significantly higher than those in LTBI, although the specificity was modest (14).

Similarly, although third-generation QFT achieved a sensitivity of 74.6% and specificity of 76.5%, it could be merely useful as an adjunct tool for the diagnosis of ATB (15). Additionally, in a study from Thailand, a high-burden country for TB, IFN-γ levels measured by QFT were higher in both ATB and LTBI groups compared with healthy controls, but could not be used alone to distinguish active disease from latent infection (16). Consequently, IGRA alone is considered unreliable for distinguishing between these disease states (17–19).

Nevertheless, several reports have suggested the potential diagnostic value of IGRA under specific conditions. For example, according to a report from France (20), counts exceeding 100 IFN-γ-secreting cells per 250,000 PBMCs were more frequently observed in the ATB group than in the LTBI group. Ledesma et al. demonstrated that the relative risk of progression to ATB increases with higher IGRA levels (21). Using the QuantiFERON test and taking 0 IU/mL as the reference, an IFN-γ concentration >20 IU/mL was associated with a relative risk of 22.31 (95% CI, 15.43–33.00). Taken together, the literature presents conflicting evidence, and the role of IGRA in distinguishing ATB from LTBI remains controversial.

In our study, we focused on elderly populations because the numbers of absolute CD4+ cells and their proportion in peripheral blood are greatly reduced compared with young adults and children (22–24). Beyond this numerical decline, the functional capacity of CD4+ T cells also diminishes with age due to thymic involution, loss of CD28 expression, and telomere shortening, leading to reduced proliferative and cytokine-producing ability (25). Thus, older individuals experience both quantitative and qualitative impairments of CD4+ T cells, which may underlie the reduced responsiveness to IGRA observed in this age group. In the study by Tebruegge et al., the rate of indeterminate QFT results was significantly higher in elderly individuals (≥65 years; 7.4%) compared with adults aged 18–64 years (2.6%) and was also elevated in children and adolescents (9.1%) (*P* < 0.0001) (26). However, a study from Japan reported that QFT-Plus remains effective for detecting active TB even in the elderly, with quantitative values significantly higher than those in LTBI or non-TB cases (27). These contrasting findings highlight the mixed evidence regarding IGRA performance in older adults, underscoring the need for further evaluation using real-world data. In our study population, quantitative values of both T-SPOT and QFT-Plus in ATB patients were generally comparable to those in LTBI patients, but both assays tended to show higher quantitative values among IGRA-positive individuals. The sensitivity and specificity for distinguishing ATB from LTBI were modest for T-SPOT according to the ROC analysis. Notably, among T-SPOT-positive patients, there was substantial variability in discriminatory performance between the two facilities.

In this study, the median quantitative T-SPOT score was higher in LTBI patients, and the positivity rate among ATB patients was considerably lower in FHUH. FHUH is a university hospital rather than a TB referral center, whereas TGH is a community hospital with dedicated TB wards. Previous studies have shown that the IGRA sensitivity can be reduced even in patients with ATB when they are in an immunosuppressed state or in those with smear-negative disease, which is often associated with a lower bacterial load (28, 29). FHUH, as a tertiary referral center, manages a larger number of immunocompromised or post-transplant patients compared with TGH, which may partly explain the reduced IGRA reactivity among ATB cases at this institution. However, because detailed patient-level background data were not available, this remains a limitation of the present study. According to Yamasue et al., factors associated with false-negative IGRA results in ATB include advanced age, peripheral lymphopenia, HIV infection, central nervous system TB, and various immunosuppressive conditions or comorbidities, such as diabetes, malignancy, chronic kidney disease, corticosteroid, or immunosuppressant use, as well as possible effects of poor nutritional status (8). In this context, the observed differences between FHUH and TGH may reflect variations in patient selection, pre-test probability, and the clinical context in which IGRA testing was performed. In our data, its ability to distinguish ATB from LTBI was overall modest but variable, suggesting that IGRA performance may vary depending on patient characteristics and disease presentation.

We acknowledge several limitations in our study. First, this study did not account for patient background factors, such as immunosuppressive status, bacterial load, or the specific indication for IGRA testing, nor did it assess CD4, CD8, or total lymphocyte counts. Detailed information on baseline comorbidities and use of immunosuppressive agents could not be obtained because the microbiology database was not directly embedded in the electronic medical record systems at either institution. However, in both institutions, IGRA testing was generally performed in accordance with national guidelines—primarily as pre-screening for organ transplantation, preoperative evaluation, or before initiating chemotherapy or immunosuppressive therapy, rather than for diagnosing active TB. In Japan, IGRA is generally recommended before initiating immunosuppressive therapy for patients with recent TB exposure, and as a supplementary test for fever of unknown origin (30). Therefore, although detailed clinical backgrounds were unavailable, the present findings reflect the overall testing performance in patient populations who underwent IGRA as part of guideline-based clinical practice.

Second, classification of ATB and LTBI was based on the presence or absence of mycobacterial testing within ±90 days of IGRA measurement. Although this time window was chosen to balance the need for temporal relevance with practical feasibility, it is possible that some patients classified as LTBI might have developed ATB outside of this window, or that certain ATB cases had microbiological confirmation beyond the 90-day period. Extending the window further, however, would have increased the risk of misclassification due to unrelated future disease events.

### Conclusion

In this study of elderly individuals in Japan, a historically high TB burden country, both T-SPOT and QFT-Plus demonstrated only modest ability to distinguish ATB from LTBI, with considerable overlap in quantitative values. Median T-SPOT scores were unexpectedly higher in LTBI than ATB in one facility, and QFT-Plus also failed to show a clear separation between groups. Notably, however, when analyses were restricted to IGRA-positive individuals, quantitative values showed a clearer trend toward differentiating ATB from LTBI, suggesting potential utility in this subset. These findings align with previous reports showing limited overall discriminatory power of IGRA and support the view that performance can vary depending on patient characteristics, disease presentation, and testing context. Taken together, our data indicate that quantitative IGRA alone cannot serve as a universal diagnostic discriminator in the elderly; however, under specific clinical conditions and with appropriate contextual interpretation, it may still contribute meaningfully to the diagnostic process.
